# ZDWX-25, a dual DYRK1A/GSK3β inhibitor, attenuates tau hyperphosphorylation and neuroinflammation in acute mouse models

**DOI:** 10.3389/fphar.2026.1892218

**Published:** 2026-08-27

**Authors:** Weiyi Wang, Yu Xing, Zonghe Xu, Siyuan Liu, Lijun Zhou, Xinyue Ning, Xinzhu Li, Fangyuan Zheng, Aizhu Yang, Zhenshu Li, Mengyu Ren, Zihua Xu, Qingchun Zhao

**Affiliations:** Department of Pharmacy, General Hospital of Northern Theater Command, Shenyang, China

**Keywords:** Alzheimer’s disease, DYRK1A, GSK3β, neuroinflammation, tau

## Abstract

**Objective:**

This study investigates the mechanisms of ZDWX-25, a novel dual DYRK1A/GSK3β inhibitor, in alleviating tau pathology and explores its potential anti-neuroinflammatory effects.

**Methods:**

Acute tauopathy was induced by okadaic acid (OKA) and neuroinflammation by lipopolysaccharide (LPS) in mice. Cognitive function was assessed by Morris water maze and Y-maze (OKA only). Tau phosphorylation (AT8), neuronal integrity (Nissl, PSD95), microglial activation (IBA-1), and inflammatory mediators were measured. The PI3K inhibitor LY294002 was used in HT22 and BV2 cells.

**Results:**

In the OKA model, ZDWX-25 improved spatial and working memory, reduced tau hyperphosphorylation, and protected neurons. It activated PI3K/AKT; LY294002 completely blocked the GSK3β inhibitor AR-A014418, whereas both the DYRK1A inhibitor harmine and ZDWX-25 retained significant, albeit reduced, efficacy, revealing direct (PI3K/AKT-independent) GSK3β inhibition plus indirect (DYRK1A→PI3K/AKT) enhancement. ZDWX-25 also promoted microglial tau phagocytosis. In the LPS model, ZDWX-25 suppressed microglial activation and inflammatory mediators (IL-6, TNF-α, iNOS) and ameliorated cognitive deficits.

**Conclusion:**

ZDWX-25 attenuates tau pathology via dual direct/indirect mechanisms and enhances microglial clearance; it also shows preliminary anti-neuroinflammatory effects. These findings support ZDWX-25 as a multi-target candidate for Alzheimer’s disease.

## 1 Introduction

Alzheimer’s disease (AD) is a neurodegenerative disorder characterized primarily by progressive memory and cognitive decline and represents the leading cause of dementia (World Health Organization, 2023). With the global AD patient population exceeding 55 million in 2022 and projected to rise to 139 million by 2050, the disease imposes a growing burden worldwide (Lonnemann et al., 2020). Its core pathological features include extracellular amyloid-β (Aβ) plaques, neurofibrillary tangles (NFTs) composed of hyperphosphorylated tau protein, and chronic neuroinflammation (Scheltens et al., 2021). Despite extensive research, no effective therapies capable of halting or reversing the progression of AD have yet emerged. The recent approval of the Aβ antibody drug Lecanemab by the FDA has advanced, yet its associated adverse events underscore the urgent need for safer treatment options (van Dyck et al., 2023). The repeated setbacks in drug development programs targeting Aβ highlight the pressing necessity of exploring alternative strategies.

Critically, the severity of tau pathology is closely correlated with cognitive impairment, whereas neuroinflammation persists throughout the disease course and is similarly associated with cognitive decline (Gomez-Isla et al., 1997; Bettcher et al., 2021). Consequently, targeting tau pathology and neuroinflammation has emerged as a promising therapeutic avenue for AD. Tau, a microtubule-associated protein, becomes hyperphosphorylated in AD, leading to its aggregation into NFTs, microtubule depolymerization, and neuronal loss (Strang et al., 2019). Moreover, pathogenic tau exhibits propagative capacity, as NFTs released from neurons can be taken up by healthy neighbors, resulting in new pathologies and driving disease progression (Holmes et al., 2014). Neuroinflammation, which is mediated primarily by glial cells, represents another pervasive AD mechanism (Jagust, 2018). In response to stimuli, microglia and astrocytes trigger neuroinflammatory responses, compromising neuronal protection and exacerbating damage (Fakhoury, 2018). This process involves dysregulated microglial polarization: an initial anti-inflammatory M2 phenotype that releases IL-10 or TGF-β dynamically shifts to a proinflammatory M1 state (Dursun et al., 2015), which is typically accompanied by NF-κB pathway upregulation and the release of cytokines, including IL-1β, IL-6, and TNF-α (Zhang and Jiang, 2015).

The kinases GSK-3β and DYRK1A are pivotal regulators of these pathological processes. GSK-3β, a serine/threonine kinase with abnormally elevated activity in the AD brain, promotes tau phosphorylation at multiple sites (e.g., Ser396, Ser202/Thr205, and Ser404) and facilitates NFT formation (Pei et al., 1997; Cai et al., 2012). Moreover, GSK-3β serves as a key regulator of neuroinflammation, activating microglia and promoting inflammatory cytokine release (Hu et al., 2017; Beurel et al., 2015). Inhibiting GSK-3β suppresses LPS-induced NF-κB activation and reduces downstream inflammatory factors *in vivo* and *in vitro* (Poonasri et al., 2023; Ajmone-Cat et al., 2016; Deng et al., 2012). Similarly, the serine/threonine kinase DYRK1A, whose overexpression is linked to early-onset AD in Down syndrome, is aberrantly upregulated in AD (Ryoo et al., 2008). DYRK1A phosphorylates multiple serine/threonine sites on tau, promoting its aggregation (Shi et al., 2008). Furthermore, DYRK1A inhibition has anti-neuroinflammatory effects, mitigating neuronal apoptosis and inflammation in mouse models (Yong et al., 2023), potentially by blocking TLR4/NF-κB pathway activation (He et al., 2023; Ju et al., 2022; Zhang et al., 2025). Critically, GSK-3β and DYRK1A play a coregulatory role in neuroinflammation, primarily through their ability to activate the NF-κB signaling pathway. This common downstream regulatory mechanism, combined with their direct and independent contributions to tau hyperphosphorylation, positions them as dual drivers of the core pathologies of AD. Therefore, the simultaneous inhibition of DYRK1A and GSK-3β represents a strategic therapeutic approach to achieve coordinated suppression of both tau pathology and neuroinflammation, offering a more comprehensive and potentially more potent and comprehensive effect than single-target strategies.

Our research team previously demonstrated that the dual GSK-3β/DYRK1A inhibitor ZDWX-25 reduces tau hyperphosphorylation and improves cognitive function in chronic APP/PS1/Tau triple transgenic mice (Liu et al., 2023). However, its detailed signaling mechanisms and potential anti-neuroinflammatory effects remained unclear. Here, we used two acute mouse models—okadaic acid (OKA)-induced tauopathy and lipopolysaccharide (LPS)-induced neuroinflammation—to address these questions. We show that ZDWX-25 attenuates tau hyperphosphorylation through a dual mechanism: direct (PI3K/AKT-independent) GSK3β inhibition together with indirect (DYRK1A→PI3K/AKT) enhancement, and that it also promotes microglial clearance of tau aggregates. In addition, ZDWX-25 exhibits preliminary anti-neuroinflammatory activity by suppressing the DYRK1A/GSK3β/NF-κB axis. Together with our previous chronic model validation, these findings support ZDWX-25 as a promising multi-target candidate for AD.

## 2 Methods

### 2.1 Animals

Male C57BL/6J mice (weighing 22 ± 2 g) were supplied by Liaoning Changsheng Biotechnology Co., Ltd. The mice were housed in a specific pathogen-free facility under standard conditions (12-h light/dark cycle, temperature of 22 °C ± 1 °C, and humidity of 50% ± 10%) with free access to a standard laboratory rodent diet and water. All animal experiments were performed in strict compliance with the National Institutes of Health Guide for the Care and Use of Laboratory Animals, and the protocol was approved by the Laboratory Animal Ethics Committee of Northern Theater Command General Hospital [Approval No. 2021(007)].

### 2.2 Experimental design and drug administration

#### 2.2.1 OKA-induced tauopathy model

**FIGURE 1:**
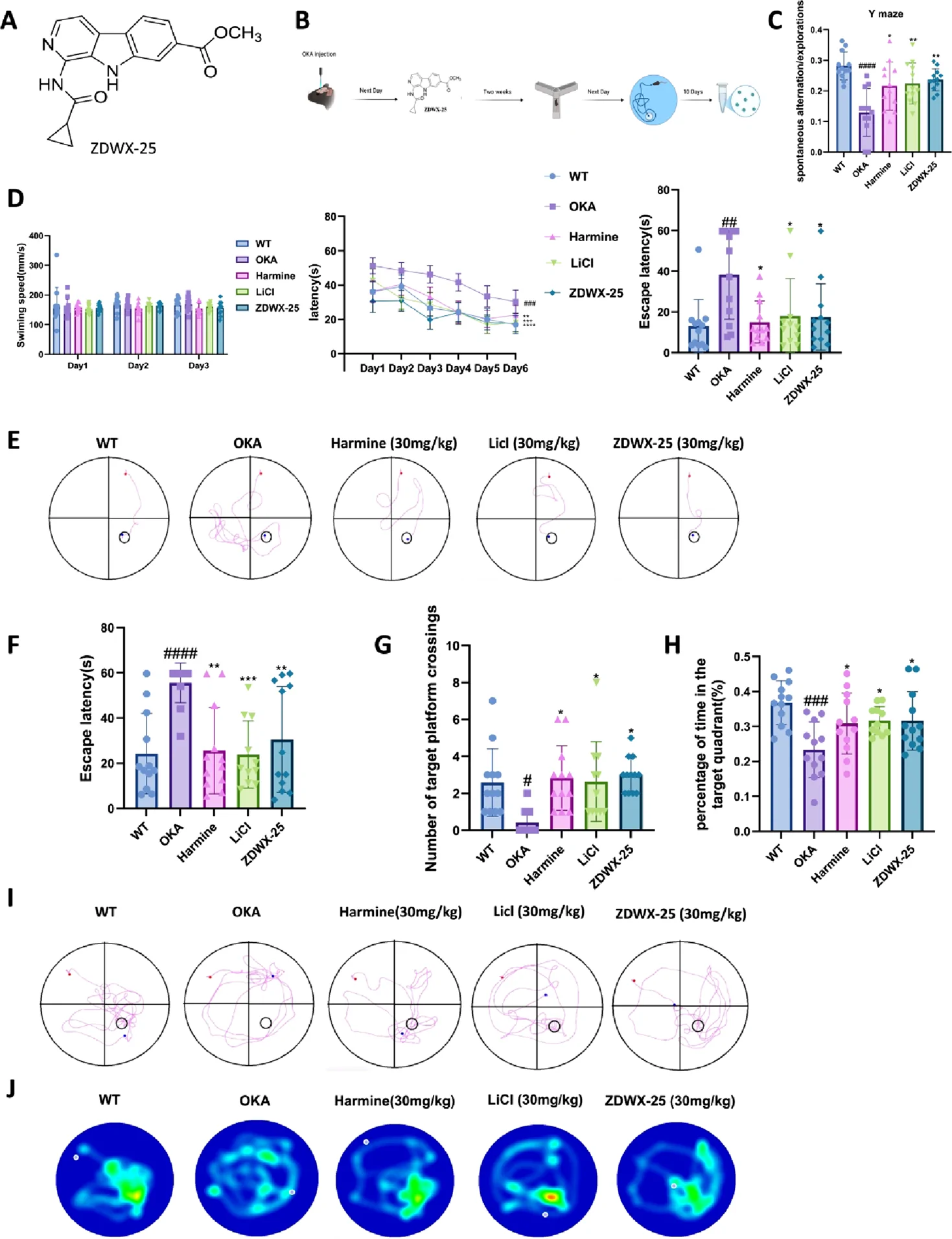
ZDWX-25 Improves Cognitive and Memory Function in OKA-Induced Acute AD Mice. **(A)** Chemical structure of ZDWX-25. **(B)** Diagram illustrating the experimental design for the *in vivo* studies. **(C)** Y-maze spontaneous alternation test: (left) representative track plots, (right) quantification of alternation percentage. **(D)** Swimming speed during the visible platform training period (days 1–3) and Escape latency during the hidden platform training sessions (days 4–9). **(E)** Representative swimming trajectories of the mice on day 7 of the hidden platform training. **(F)** Escape latency of the mice during the probe trial (spatial exploration phase). **(G)** Number of platform crossings during the probe trial. **(H)** Representative swimming trajectories during the probe trial. **(I)** Regional heatmap of time spent in the pool during the probe trial. The data are presented as the means ± SEMs (n = 12). ^#^p < 0.05, ^##^p < 0.01, ^###^p < 0.001, ^####^p < 0.001 vs. the WT group; ^*^p < 0.05, ^**^p < 0.01, ^***^p < 0.001, ^****^p < 0.0001 vs. the Model group.

A total of 60 mice were randomly divided into five experimental groups (n = 12 per group): (1) the wild-type (WT) control group; (2) the OKA model group; (3) the Harmine (30 mg/kg, DYRK1A inhibitor) treatment group; (4) the LiCl (30 mg/kg, GSK-3β inhibitor) treatment group; and (5) the ZDWX-25 (30 mg/kg) treatment group. The chemical structure of ZDWX-25 is shown in Figure 1A. The dosing regimens for harmine, LiCl, and ZDWX-25 were selected on the basis of our previous studies, which demonstrated both efficacy and safety profiles (Liu et al., 2023). To induce acute tauopathy, the mice were anesthetized and administered a single intracerebroventricular injection of OKA (100 ng in 2 μL vehicle) into the left lateral ventricle via stereotaxic coordinates (0.5 mm posterior to bregma, 1.1 mm lateral, 3.0 mm deep). Drug treatments were administered orally once daily for 14 consecutive days, commencing immediately after surgery.

#### 2.2.2 LPS-induced neuroinflammation model

For the neuroinflammation study, 42 mice were randomly allocated into six groups (n = 7 per group): (1) the WT control group; (2) the LPS model group; (3–5) the ZDWX-25 treatment groups at low, medium, and high doses (5, 10, and 20 mg/kg, respectively); and (6) the galantamine (4 mg/kg) positive control group. The dosing regimens for ZDWX-25 and the positive control galantamine, as well as the LPS challenge dose, were selected on the basis of established protocols in the literature (Liu et al., 2018; Bao et al., 2020). All the compounds were administered orally for 13 days. Starting from day 7, neuroinflammation was induced via intraperitoneal (i.p.) injections of LPS (1 mg/kg) for 6 days.

### 2.3 Y maze

The Y-maze test was performed to assess short-term spatial working memory 14 days after drug treatment. The apparatus consisted of a black polycarbonate maze with three symmetrical arms (40 cm × 8 cm × 15 cm each) radiating from a central hub (10 cm in diameter) at 120° angles. Before the test, mice were individually housed in the behavioral testing room for 24 h for acclimatization. During the test, the three arms were designated as A, B, and C using the VisuTrack animal behavior analysis system (V3.0, Shanghai Xinsoft). Each arm was assigned a distinct visual cue (circle, triangle, or square) at its far end to aid spatial orientation. Mice were placed at the distal end of a randomly chosen start arm (counter-balanced across animals) facing the center, and allowed to explore the maze freely for 5 min. The system automatically recorded the movement trajectory and the sequence of arm entries. An arm entry was counted only when the whole body (including the tail) crossed the entrance line. The total number of arm entries (i.e., the total number of times the mouse entered any arm, with consecutive entries into the same arm counted as one) and the number of spontaneous alternations (i.e., entries into three different arms consecutively, e.g., A→B→C or C→A→B) were recorded. The alternation percentage was calculated as:

$$\text{Alternation }\left(\%\right)=\left[\text{spontaneous alternations }/\;\left(\text{total arm entries}-2\right)\right]\;*\;100\%.$$

### 2.4 Morris water maze

The Morris water maze test was employed to evaluate spatial learning and memory in mice (Sun et al., 2021; Vorhees and Williams, 2006). Briefly, the test consisted of a 9-day training protocol. The first 3 days involved visible platform training to acclimate the mice to the task. During the subsequent 6-day hidden platform phase, the platform was submerged, and spatial cues were utilized for navigation. On day 9, an escape latency test was conducted. A probe trial was performed on day 10, wherein the platform was removed, and the swimming trajectory, time spent in the target quadrant, and number of crossings over the former platform location were recorded over 60 s to assess spatial memory.

### 2.5 Tissue collection and processing

Following behavioral testing, blood was collected, and serum was isolated by centrifugation at 3,000 × g for 10 min and stored at −80 °C. A subset of mice (n = 3 per group) was transcardially perfused with saline followed by 4% paraformaldehyde (PFA). The brains were postfixed in 4% PFA for 24 h and processed for paraffin sectioning. For biochemical analyses, cortical and hippocampal tissues from the remaining mice were rapidly dissected on ice, snap-frozen in liquid nitrogen, and stored at −80 °C.

### 2.6 Histological and immunofluorescence staining

#### 2.6.1 Nissl staining

Paraffin-embedded brain sections (4 μm thick) were deparaffinized, rehydrated, and stained with 0.1% cresyl violet solution (Servicebio, China) for 8–10 min at 37 °C according to standard protocols. After being differentiated in acetic ethanol, the sections were dehydrated through a graded ethanol series, cleared in xylene, and mounted with neutral balsam. Neuronal morphology and density in the hippocampal CA1, CA3, and DG subfields were examined under a light microscope (Nikon, Japan). Viable neurons, characterized by large, clear nuclei and abundant Nissl substances, were quantified from three randomly selected, nonoverlapping fields per section by an investigator blinded to the experimental groups.

#### 2.6.2 Immunohistochemistry

For the detection of microglia, the sections were subjected to antigen retrieval by heating in citrate buffer (pH 6.0) and then incubated with 3% hydrogen peroxide to quench endogenous peroxidase activity. After being blocked with 5% bovine serum albumin (BSA), the sections were incubated overnight at 4 °C with a primary antibody against IBA-1 (1:500 dilution; Servicebio, GB12105). The sections were subsequently incubated with a horseradish peroxidase (HRP)-conjugated goat anti-rabbit secondary antibody (Servicebio) at room temperature for 50 min. Immunoreactivity was visualized via a 3,3′-diaminobenzidine (DAB) substrate kit (Servicebio), and the sections were counterstained with hematoxylin. IBA-1-positive microglia in the hippocampus were imaged and quantified.

#### 2.6.3 Immunofluorescence

For double-labeling immunofluorescence, brain sections were subjected to antigen retrieval and blocked with 5% BSA. The sections were then incubated overnight at 4 °C with a mixture of primary antibodies, including mouse anti-phospho-Tau (AT8, Ser202/Thr205, 1:200 dilution, Selleck, F0059) and rabbit anti-PSD95 (1:300 dilution, Affinity, AF7746). After washing, the sections were incubated for 1 h at room temperature in the dark with a mixture of Alexa Fluor 488-conjugated goat anti-mouse and Alexa Fluor 594-conjugated goat anti-rabbit secondary antibodies (1:400, Servicebio). The cell nuclei were counterstained with DAPI (Servicebio).

### 2.7 Cell culture

#### 2.7.1 Cell lines and culture conditions

HT22 mouse hippocampal neurons (Puno Sai, CL-0697). BV2 mouse microglia (Puno Sai, CL-0493). Both cell lines were cultured in DMEM supplemented with 10% FBS and 1% penicillin/streptomycin at 37 °C in a 5% CO_2_ atmosphere.

#### 2.7.2 *In vitro* tauopathy model

Brain tissues from APP/PS1/Tau triple-transgenic AD mice (3xTg) (Beijing HUAFUKANG Bioscience Co. Inc.) were placed in a centrifuge tube containing 1 mL of PBS, two large grinding steel beads, and one small grinding steel bead. The tissue was thoroughly homogenized via a homogenizer. Following homogenization, the sample was centrifuged at 13,500 rpm for 10 min at 4 °C. The resulting supernatant was then carefully collected, transferred to a new EP tube, and stored at −80 °C for subsequent use in the BV2 phagocytosis assay.

HT22 cells were treated with 40 nM OKA for 24 h to induce tau hyperphosphorylation. Concurrently, the cells were treated with 3 μM harmine, AR-A014418, or ZDWX-25. For pathway inhibition, the PI3K inhibitor LY294002 (10 μM) was added 2 h prior to OKA.

BV2 Phagocytosis Model: BV2 cells were pretreated with 3 μM of the indicated drugs (harmine, AR-A014418, or ZDWX-25) for 2 h, followed by stimulation with 10 μL of the prepared APP/PS1/tau mouse brain homogenate for 6 h to assess phagocytic activity. To obtain conditioned medium (CM) for subsequent neuronal treatment, BV2 cells were first stimulated with the brain homogenate for 6 h and then treated with the drugs for an additional 18 h. The CM was collected, centrifuged to remove cellular debris, and then applied to naïve HT22 cells for 24 h to evaluate its effects on tau aggregation.

#### 2.7.3 *In vitro* neuroinflammation model

BV2 cells were pretreated with ZDWX-25 (0.3, 1, or 3 μM) or galantamine (20 μM) for 2 h, followed by stimulation with LPS (1 μg/mL) for 24 h. The resulting CM was transferred to HT22 cells for another 24 h of culture.

### 2.8 Cell viability assay (MTT)

Cell viability was determined via the MTT assay. The cells were seeded in 96-well plates (8 × 10^3^ cells/well). After treatment, MTT solution (5 mg/mL) was added to each well and incubated for 4 h. The resulting formazan crystals were dissolved in DMSO, and the absorbance was measured at 490 nm.

### 2.9 Detection of tau aggregates in HT22 and BV2 cells

The pyrazine-quinoxaline probe QNN-3b specifically labels neurofibrillary tangles (NFTs) and can be used for the fluorescent detection of Tau aggregates both *in vivo* and *in vitro*. Notably, its fluorescence signal closely correlates with Tau phosphorylation at the Ser202/Thr205 epitope (Zhu et al., 2018). After the cell model was established, the culture medium was discarded. The cells were subsequently fixed with 4% paraformaldehyde for 10 min, followed by permeabilization with 0.1% Triton X-100 for 10 min. After each step, the samples were washed three times with PBS for 5 min each. Under light-protected conditions, a 10 μM QNN-3b probe (50 μL/well) was added, and the mixture was incubated for 15 min. The samples were washed three times with PBS, each for 10 min. Subsequently, DAPI (50 μL/well) was added, and the samples were stained for 15 min to label the cell nuclei. Finally, the QNN-3b red fluorescence signal was observed under a fluorescence microscope. The QNN-3b probe was provided by Professor Cheng Yan from Sichuan University.

### 2.10 Western blot and ELISA

Western blot analysis was conducted as previously described (Liu et al., 2021). Briefly, protein lysates from tissues or cells were separated via SDS‒PAGE, transferred to PVDF membranes, and incubated overnight at 4 °C with the following primary antibodies: p-AKT (Ser473; HuaBio, ET1607-73), AKT (HuaBio, ET1609-47), p-GSK-3β (Ser9; Affinity, #AF2016), GSK-3β (Affinity, #AF5016), DYRK1A (ABclonal, A0595), IκBα (HuaBio, ET1603-6), p-IκBα (HuaBio, ET1603-2), NF-κB p65 (HuaBio, HA500402), p-NF-κB p65 (HuaBio, ET1604-27), iNOS (Affinity, #AF0199), COX-2 (Wanlei, WL01750), TNF-α (Wanlei, WL01581), Caspase-3/cleaved Caspase-3 (Wanlei, WL02117), Bcl-2 (HuaBio, HA721235), GAPDH (Affinity, #AF7021), and β-Actin (Affinity, #AF8018). Both anti-mouse and anti-rabbit immunoglobulin G (IgG) horseradish peroxidase -conjugated antibodies were obtained from HUABIO (China). The protein bands were visualized via a Bio-Rad ChemiDoc MP Imaging System and quantified via ImageJ software.

The levels of TNF-α and IL-6 in the cell culture supernatants and mouse serum were measured via commercial ELISA kits according to the manufacturer’s instructions.

### 2.11 NO detection

NO production was assessed by measuring nitrite accumulation in supernatants via the Griess reaction. The samples were mixed with Griess reagents and incubated for 10 min, after which the absorbance was measured at 540 nm.

### 2.12 Statistical analysis

All data are presented as the mean ± standard error of the mean (SEM). Statistical analyses were performed via GraphPad Prism 9.0. Differences between groups were analyzed by one-way or two-way analysis of variance (ANOVA) followed by Tukey’s or Bonferroni *post hoc* correction. A value of *p < 0.05 was considered statistically significant.

## 3 Results

### 3.1 ZDWX-25 significantly improved cognitive and memory function in OKA-induced acute tauopathy mice

To investigate the effects of ZDWX-25 in tau-pathological mice, we established a mouse model of tau hyperphosphorylation via intraventricular injection of OKA. After 14 days of oral administration, cognitive function was assessed via the Y Maze and MWM test (Figure 1B). In the Y-maze spontaneous alternation test, OKA-induced model mice exhibited a significantly lower alternation percentage compared with WT controls, indicating impaired working memory. In contrast, all drug-treated groups showed increased alternation rates, with the ZDWX-25 group demonstrating the most pronounced effect (Figure 1C).

In the MWM test, during the initial 3-day visible platform training phase, no significant differences in swimming speed were observed among the experimental groups (Figure 1D). These results indicate that ZDWX-25 and OKA treatment did not affect the motor function of the mice, ensuring that the subsequent spatial learning results were not confounded by physical impairments. Compared with WT control model mice, OKA-induced model mice presented a significantly prolonged escape latency. In contrast, all the drug-treated groups presented a marked reduction in escape latency, with the ZDWX-25 group performing at a level comparable to that of the WT group (Figure 1D). On day 9 of training, representative swimming paths revealed a disorganized search strategy in the Model group, whereas mice treated with ZDWX-25 navigated more directly to the platform location (Figure 1E). In the subsequent probe trial, ZDWX-25-treated mice displayed significantly shorter escape latencies (Figure 1F) and a greater number of crossings over the former platform location (Figure 1G) than did the Model group. Heatmap analysis further corroborated these findings, indicating that the treated mice spent considerably more time in the target quadrant (Figures 1H,I). These findings collectively demonstrate that ZDWX-25 significantly enhances spatial learning and long-term memory capacity in AD mice.

### 3.2 ZDWX-25 ameliorates hippocampal and cortical tau pathology in AD model mice and protects neurons

**FIGURE 2:**
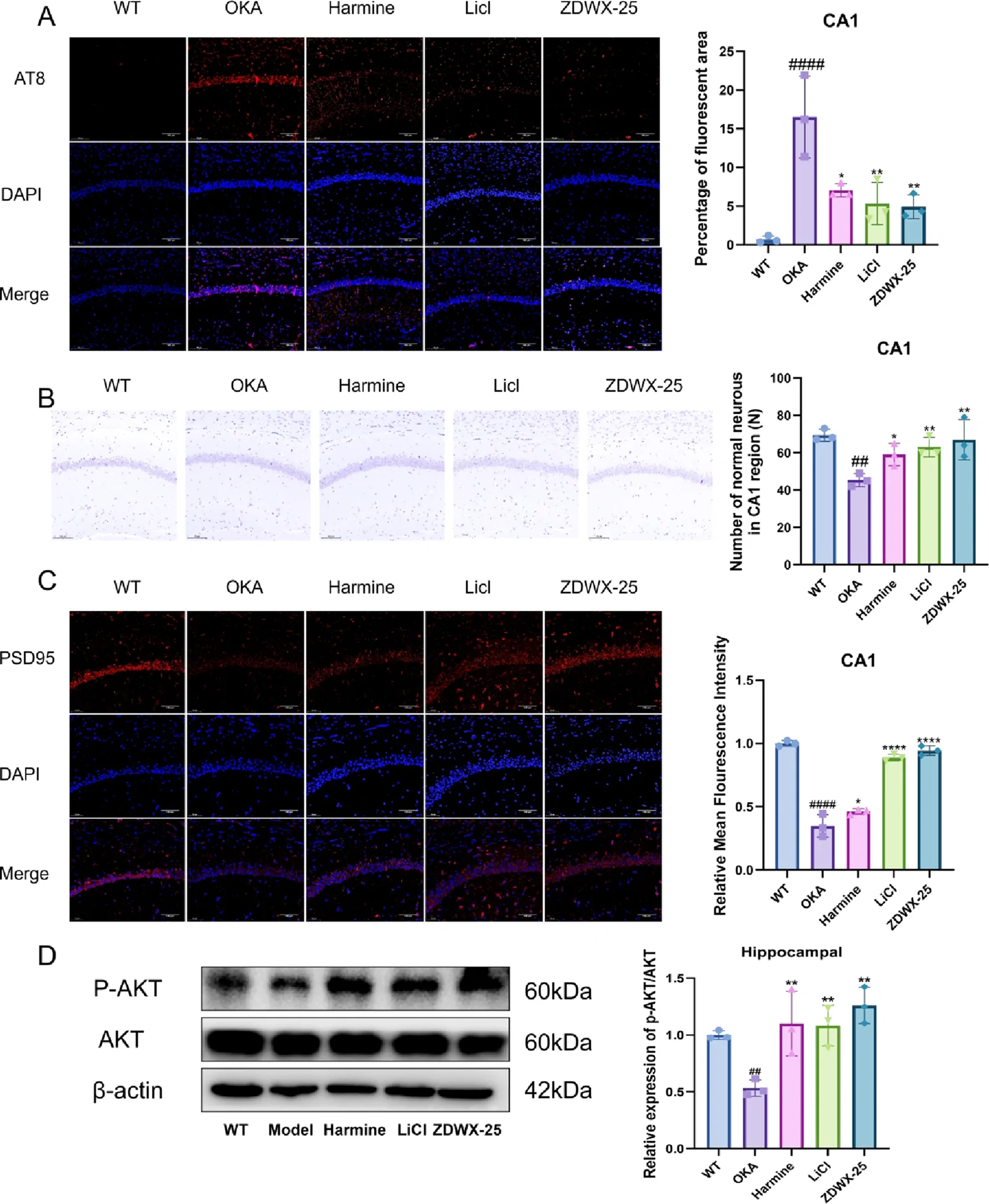
ZDWX-25 ameliorates tau pathology in the brains of AD mice and protects neurons. **(A)** AT8 immunofluorescence and statistical results in the hippocampal CA1 region of mice. (Scale bar = 100 μm). **(B)** Nissl staining and statistical results in the hippocampal CA1 region of the mice. (Scale bar = 100 μm). **(C)** PSD95 immunofluorescence and statistical results in the CA1 region of the mouse brains. (Scale bar = 100 μm). **(D)** Expression of p-AKT and AKT in the hippocampal tissue of the mice. ^##^p < 0.01, ^####^p < 0.0001 vs. the WT group; ^*^p < 0.05, ^**^p < 0.01, ^****^p < 0.0001 vs. the model group. (n = 3 means ± SEMs). This figure presents a composite of Western blot bands from the same experiment and sample set that were run in parallel on separate gels and individually exposed. All bands represent parallel experimental results from the same batch of samples and have been uniformly processed and compared.

Next, we evaluated the neuroprotective effects of ZDWX-25 against OKA-induced neuropathology. Immunofluorescence staining using the AT8 antibody (p-Tau Ser202/Thr205) revealed a pronounced increase in immunoreactivity within the hippocampal CA1 region of the OKA-treated group, confirming extensive tau hyperphosphorylation. Treatment with ZDWX-25 significantly attenuated this pathological phosphorylation (Figure 2A). Furthermore, Nissl staining showed that OKA induction led to a disorganized neuronal architecture and a substantial loss of viable neurons in the hippocampus. These morphological deficits were effectively ameliorated by ZDWX-25, as evidenced by the preserved density of Nissl-positive cells in the CA1 layer (Figure 2B). Given the pivotal role of synaptic integrity in cognitive function, we assessed the postsynaptic marker PSD95. The marked reduction in PSD95 fluorescence observed in the model group was potently reversed by ZDWX-25, indicating a protective effect on synaptic scaffolds (Figure 2C).

Considering that the PI3K/AKT signaling axis is a critical mediator of neuroprotection and tau regulation, we investigated the expression of key proteins via Western blot analysis. Compared to the WT group, the model group exhibited significantly lower p-AKT levels; however, this downregulation was markedly reversed following ZDWX-25 intervention (Figure 2D). Collectively, these results suggest that ZDWX-25 mitigates tau hyperphosphorylation and neuronal damage, potentially through the activation of the PI3K/AKT signaling pathway.

### 3.3 ZDWX-25 ameliorates OKA-induced tau hyperphosphorylation in HT-22 cells via the PI3K/AKT pathway

**FIGURE 3:**
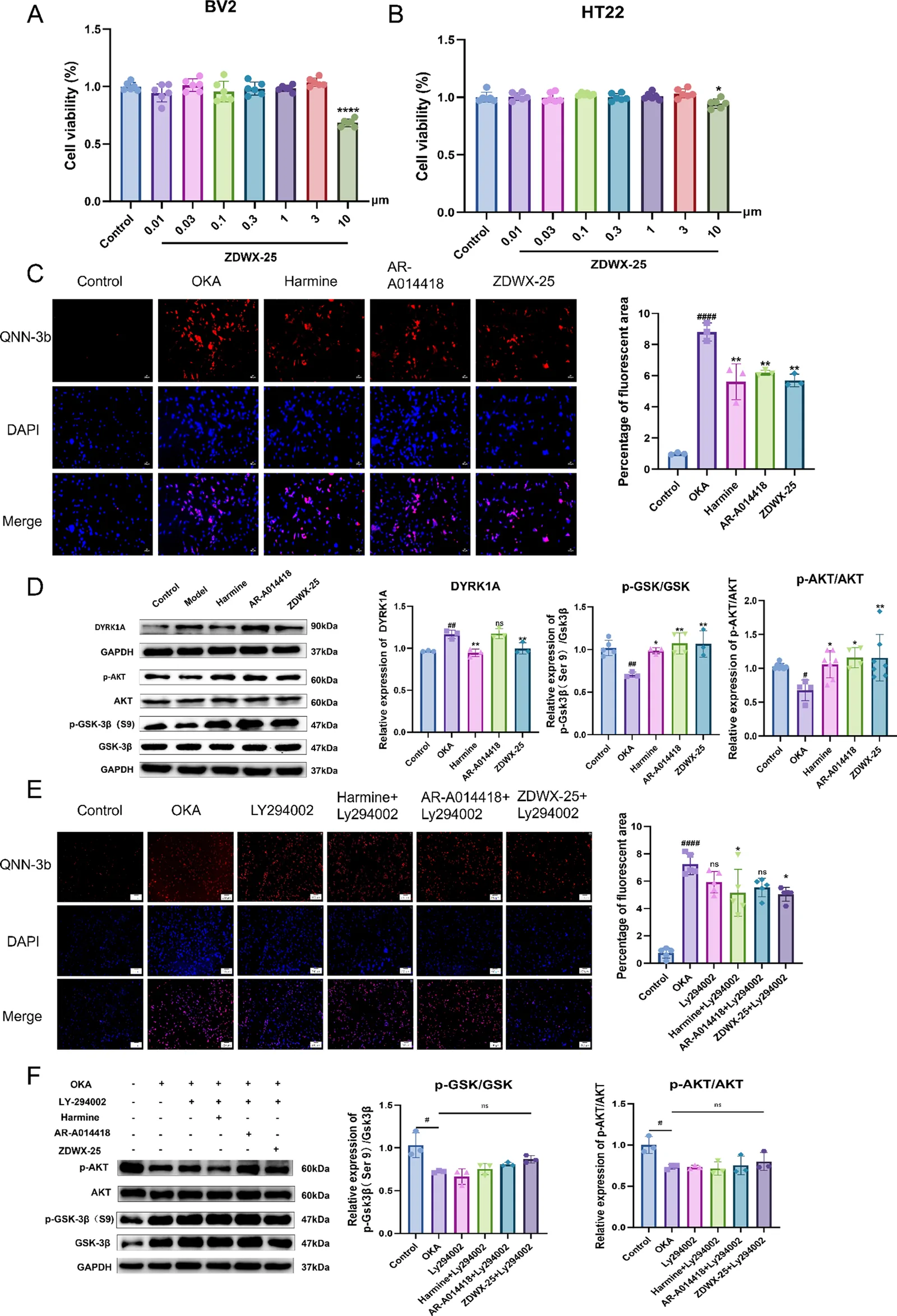
ZDWX-25 reduces OKA-induced hyperphosphorylation of tau protein in HT22 cells via the PI3K/AKT pathway. **(A)** MTT assay results for BV2 cells. **(B)** MTT assay results for HT22 cells. *p < 0.05, ****p < 0.0001 vs. the control group. **(C)** Fluorescence results of the QNN-3b probe in HT22 cells. (Scale bar = 20 μm). **(D)** Expression of DYRK1A, p-AKT, AKT, p-GSK3β, and GSK3β in HT22 cells. **(E)** QNN-3b probe fluorescence results in HT22 cells following Ly294002 pretreatment. (Scale bar = 20 μm). **(F)** Expression of DYRK1A, p-AKT, AKT, p-GSK3β, and GSK3β in HT22 cells following Ly294002 pretreatment. ^#^p < 0.05, ^##^p < 0.01, ^####^p < 0.0001 vs. the control group; ^*^p < 0.05, ^**^p < 0.01, vs. the model group. (n = 5 mean ± SEM). This figure presents a composite of Western blot bands from the same experiment and sample set that were run in parallel on separate gels and individually exposed. All bands represent parallel experimental results from the same batch of samples and have been uniformly processed and compared.

Prior to mechanistic investigation, we first evaluated the potential cytotoxicity of ZDWX-25 via the MTT assay. Treatment for 24 h revealed that ZDWX-25 did not significantly affect the viability of either HT22 hippocampal neuronal cells or BV2 microglia at concentrations up to 10 μM (Figures 3A,B). On the basis of these results, a safe and effective concentration range of 0.3–3 μM was selected for all subsequent *in vitro* experiments.

We first validated the efficacy of ZDWX-25 in reducing tau hyperphosphorylation *in vitro*. Utilizing the QNN-3b fluorescent probe, we observed that OKA stimulation significantly enhanced the fluorescence intensity in HT22 cells; however, this effect was effectively reduced by treatment with harmine (DYRK1A inhibitor), AR-A014418 (GSK-3β inhibitor), or ZDWX-25 (Figure 3C). Western blot analysis was performed to elucidate the underlying mechanism. OKA treatment inhibited the PI3K/AKT pathway, evidenced by diminished AKT phosphorylation. However, treatment with ZDWX-25 not only restored p-AKT levels but also concomitantly increased the inhibitory phosphorylation of GSK-3β at Ser9. Notably, pharmacological inhibition of DYRK1A by harmine similarly elevated both p-AKT and p-GSK-3β levels. In contrast, the GSK-3β inhibitor AR-A014418 did not alter DYRK1A expression. Furthermore, both harmine and ZDWX-25—but not AR-A014418—effectively abrogated the OKA-induced upregulation of DYRK1A (Figure 3D). Collectively, these findings suggest that DYRK1A inhibition suppresses GSK-3β activity, potentially through the activation of the PI3K/AKT signaling axis.

We investigated the involvement of the PI3K/AKT pathway using the inhibitor LY294002. While LY294002 completely abolished the protective effects of AR-A014418, both harmine and ZDWX-25 retained significant activity against OKA-induced tau hyperphosphorylation (Figure 3E). Notably, Western blot analysis showed that LY294002 fully suppressed AKT activation and GSK-3β Ser9 phosphorylation across all groups (Figure 3F), demonstrating that the residual efficacy of harmine and ZDWX-25 is independent of PI3K/AKT signaling.

Notably, AR-A014418 lost its efficacy upon PI3K blockade. In contrast, harmine’s direct inhibition of DYRK1A targets tau phosphorylation through a distinct, pathway-independent mechanism. By combining these two functionalities, ZDWX-25 leverages a dual inhibitory action that circumvents the limitations of single-target GSK3β inhibitors. These data underscore the superior therapeutic resilience of ZDWX-25, which remains effective even under conditions of impaired PI3K/AKT signaling—a hallmark of neurodegenerative pathology.

### 3.4 ZDWX-25 regulates the phagocytosis and propagation of NFTs by microglia via the PI3K/AKT signaling pathway

**FIGURE 4:**
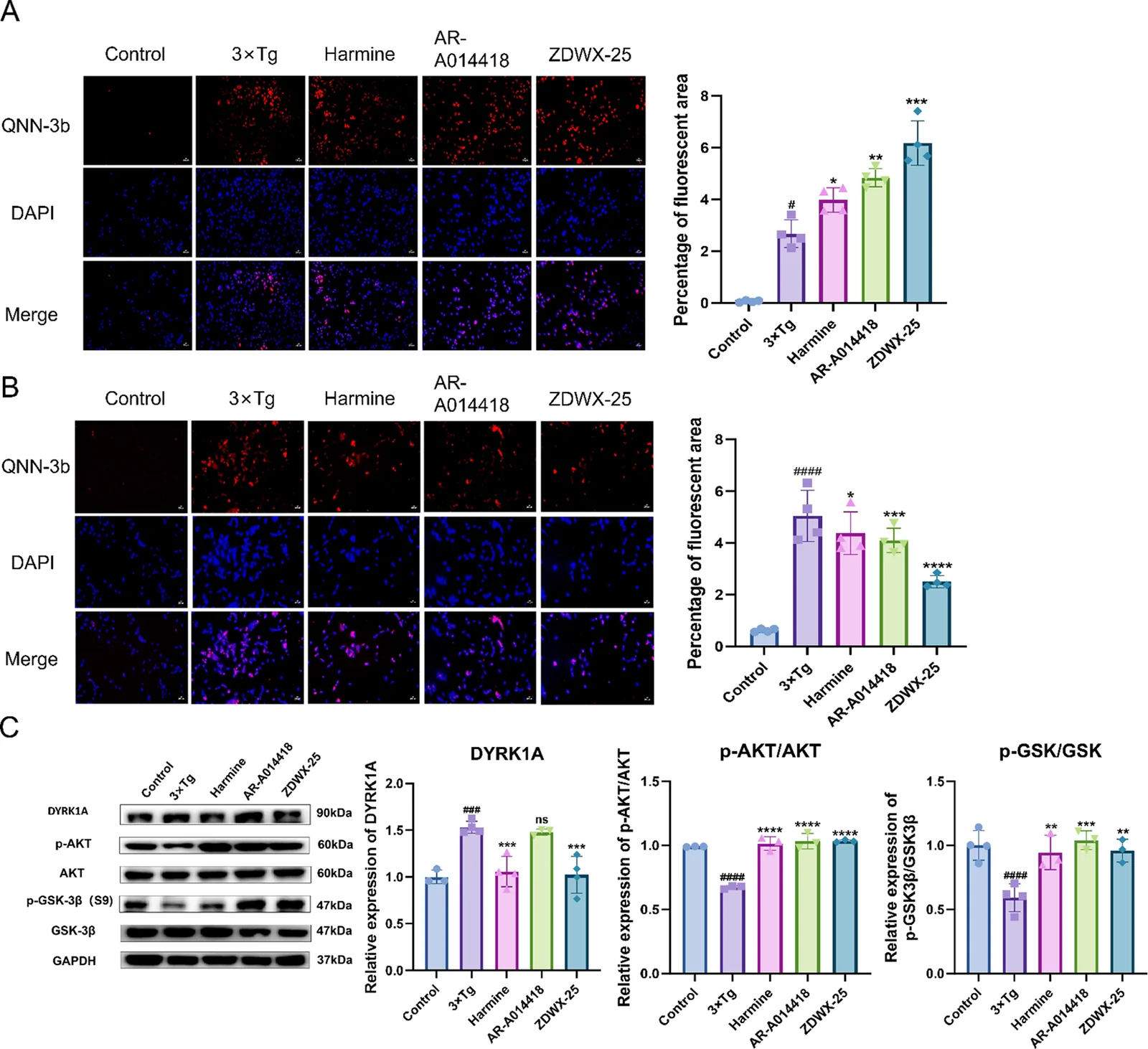
ZDWX-25 promotes the phagocytosis of NFTs by microglia and inhibits their propagation. **(A)** QNN-3b probe fluorescence results for BV2 phagocytosis of NFTs. (Scale bar = 20 μm). **(B)** QNN-3b probe fluorescence results in HT22 cells treated with BV2 -conditioned medium. **(C)** Expression of DYRK1A, p-AKT, AKT, p-GSK3β, and GSK3β in BV2 cells. ^#^p < 0.05, ^###^p < 0.001, ^####^p < 0.0001 vs. the control group; ^*^p < 0.05, ^**^p < 0.01, ^***^p < 0.001 vs. the model group. (n = 5 mean ± SEM). This figure presents a composite of Western blot bands from the same experiment and sample set that were run in parallel on separate gels and individually exposed. All bands represent parallel experimental results from the same batch of samples and have been uniformly processed and compared.

Having established the effects of ZDWX-25 on neurons, we investigated its impact on microglial function in tau pathology. Using the QNN-3b probe, we first assessed the phagocytosis of NFTs by BV2 microglia. Treatment with harmine, AR-A014418, or ZDWX-25 significantly enhanced the phagocytic capacity of BV2 cells, with ZDWX-25 exhibiting the most potent effect (Figure 4A). We next asked whether enhanced phagocytosis translates to reduced propagation of pathological tau to neurons. BV2 cells were pretreated with drugs and stimulated with brain homogenate to generate conditioned medium (CM), which was then applied to naïve HT22 neurons. Compared with CM from untreated BV2 cells, QNN-3b staining revealed that CM from drug-treated BV2 cells significantly reduced the accumulation of tau aggregates in recipient HT22 cells (Figure 4B), indicating that ZDWX-25 inhibits microglia-mediated tau propagation. Western blot analysis of BV2 cells revealed that ZDWX-25 and harmine treatment activated the PI3K/AKT pathway (increased p-AKT) and inhibited GSK-3β (increased p-GSK-3β Ser9). Furthermore, both compounds, but not AR-A014418, reduced the protein expression of DYRK1A (Figure 4C). These findings suggest that ZDWX-25 promotes microglial phagocytosis and restricts tau propagation by concurrently inhibiting DYRK1A and activating the PI3K/AKT/GSK-3β pathway.

**FIGURE 5:**
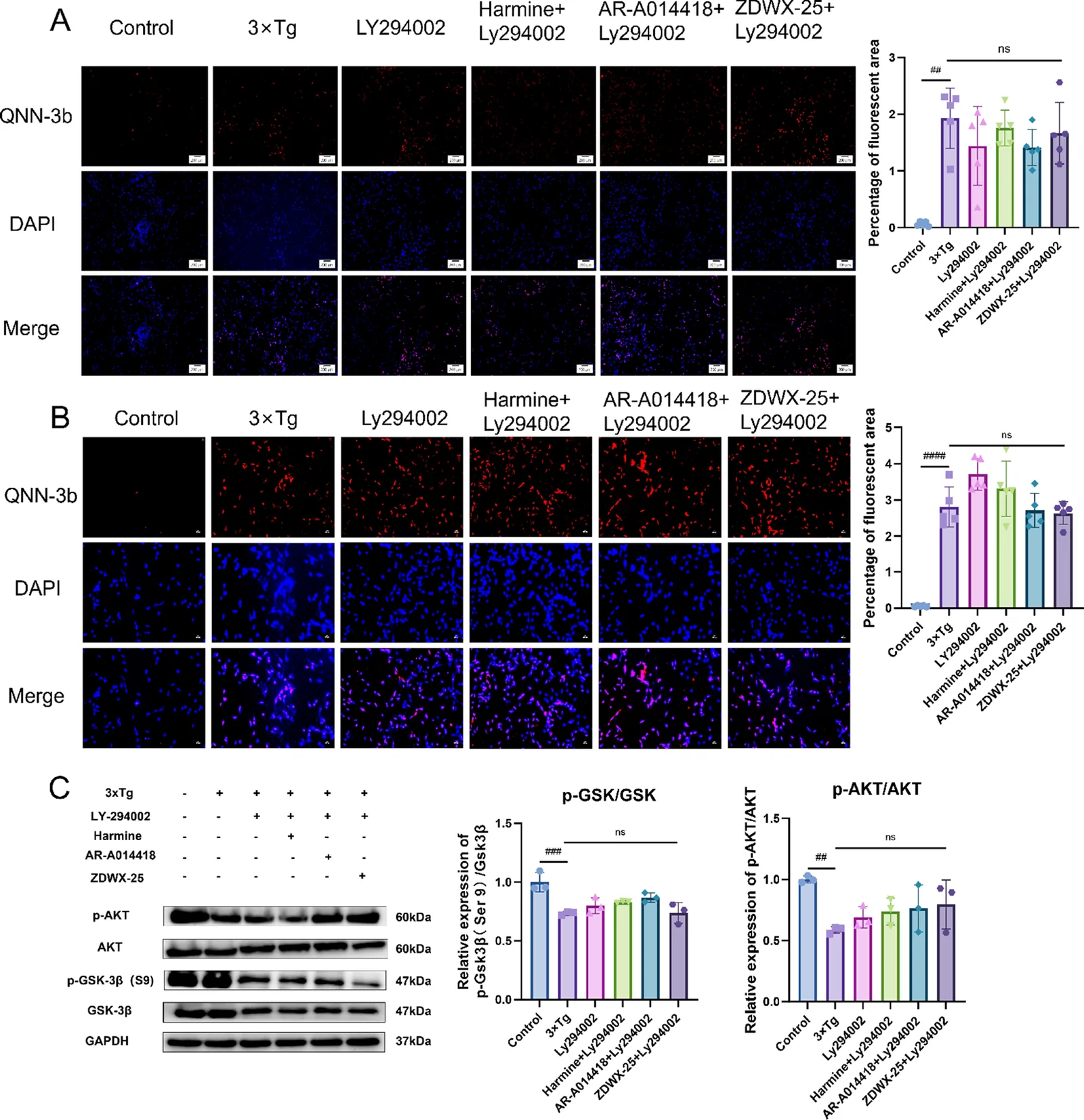
LY294002 pretreatment inhibits ZDWX-25-mediated regulation of microglial phagocytic and propagative functions. **(A)** QNN-3b probe fluorescence results for BV2 phagocytosis of NFTs following Ly294002 pretreatment. (Scale bar = 20 μm). **(B)** QNN-3b probe fluorescence results in HT22 cells treated with BV2 -conditioned medium following Ly294002 pretreatment. **(C)** Expression of DYRK1A, p-AKT, AKT, p-GSK3β, and GSK3β in BV2 cells following Ly294002 pretreatment. ^#^p < 0.01, ^###^p < 0.001, ^####^p < 0.0001 vs. the control group (n = 5 mean ± SEM). This figure presents a composite of Western blot bands from the same experiment and sample set that were run in parallel on separate gels and individually exposed. All bands represent parallel experimental results from the same batch of samples and have been uniformly processed and compared.

To confirm the central role of the PI3K/AKT pathway in mediating the effects of ZDWX-25 on microglia, we pretreated BV2 cells with the PI3K inhibitor LY294002. LY294002 completely abolished the ability of all the drugs, including ZDWX-25, to enhance NFT phagocytosis (Figure 5A). Consistently, the conditioned medium from LY294002-pretreated BV2 cells lost its ability to suppress tau aggregation in HT22 neurons, with the fluorescence intensity reverting to that of the model group (Figure 5B). At the molecular level, LY294002 pretreatment prevented the drug-induced phosphorylation of AKT and GSK-3β in BV2 cells. Notably, the regulatory effect of harm on DYRK1A, AKT, and GSK-3β was also negated by pathway inhibition (Figure 5C). These results unequivocally demonstrate that the enhancement of microglial function by ZDWX-25 is strictly dependent on the activation of the PI3K/AKT signaling pathway.

### 3.5 ZDWX-25 improves cognitive and memory function in LPS-Induced neuroinflammatory mice

**FIGURE 6:**
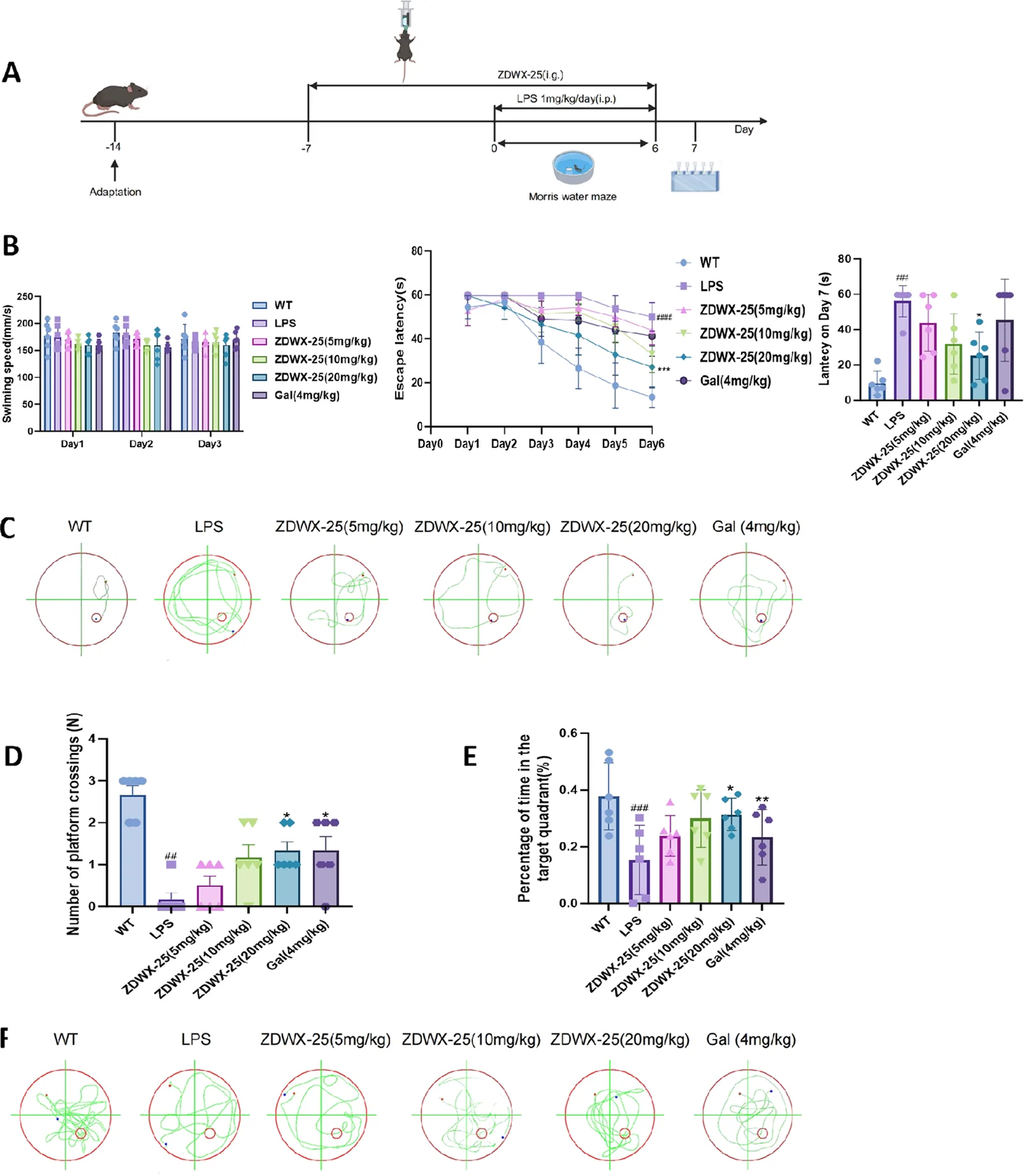
ZDWX-25 improves cognitive and memory functions in mice with LPS-induced neuroinflammation. **(A)** Diagram illustrating the experimental design for the *in vivo* studies. **(B)** Swimming speed during the visible platform training period (days 1–3) and statistical graph of escape latency. **(C)** Swimming trajectories of the mice on day 7 of escape latency testing. **(D)** Number of platform crossings during the spatial exploration phase. **(E)** Time spent in the target quadrant during the spatial exploration phase. **(F)** Behavioral trajectory diagram during the spatial exploration phase. The data are expressed as the means ± standard errors (n = 7). ^##^p < 0.01, ^###^p < 0.001, ^####^p < 0.0001 compared with the wild-type group; ^*^p < 0.05, ^**^p < 0.01, ^***^p < 0.001 compared with the model group.

The efficacy of ZDWX-25 was subsequently assessed in an LPS-induced model of neuroinflammation. In the Morris water maze test, no significant differences in swimming speed were observed among the different groups of mice during the visible platform training period on the first 3 days (Figure 6B). Compared with WT mice, LPS-treated mice exhibited significant cognitive deficits in the MWM test, with longer escape latencies during hidden platform training. ZDWX-25 treatment (20 mg/kg) significantly shortened the escape latency and improved the search strategy, as evidenced by more direct swimming paths (Figures 6B,C). In the probe trial, ZDWX-25 (20 mg/kg)-treated mice made significantly more crossings over the former platform location (Figure 6D) and spent more time in the target quadrant (Figures 6E,F) than the Model group did. These results indicate that ZDWX-25 effectively ameliorates the cognitive impairment induced by systemic neuroinflammation.

### 3.6 ZDWX-25 ameliorates LPS-induced neuroinflammation by attenuating neuronal injury, microglial activation, and proinflammatory mediator release

**FIGURE 7:**
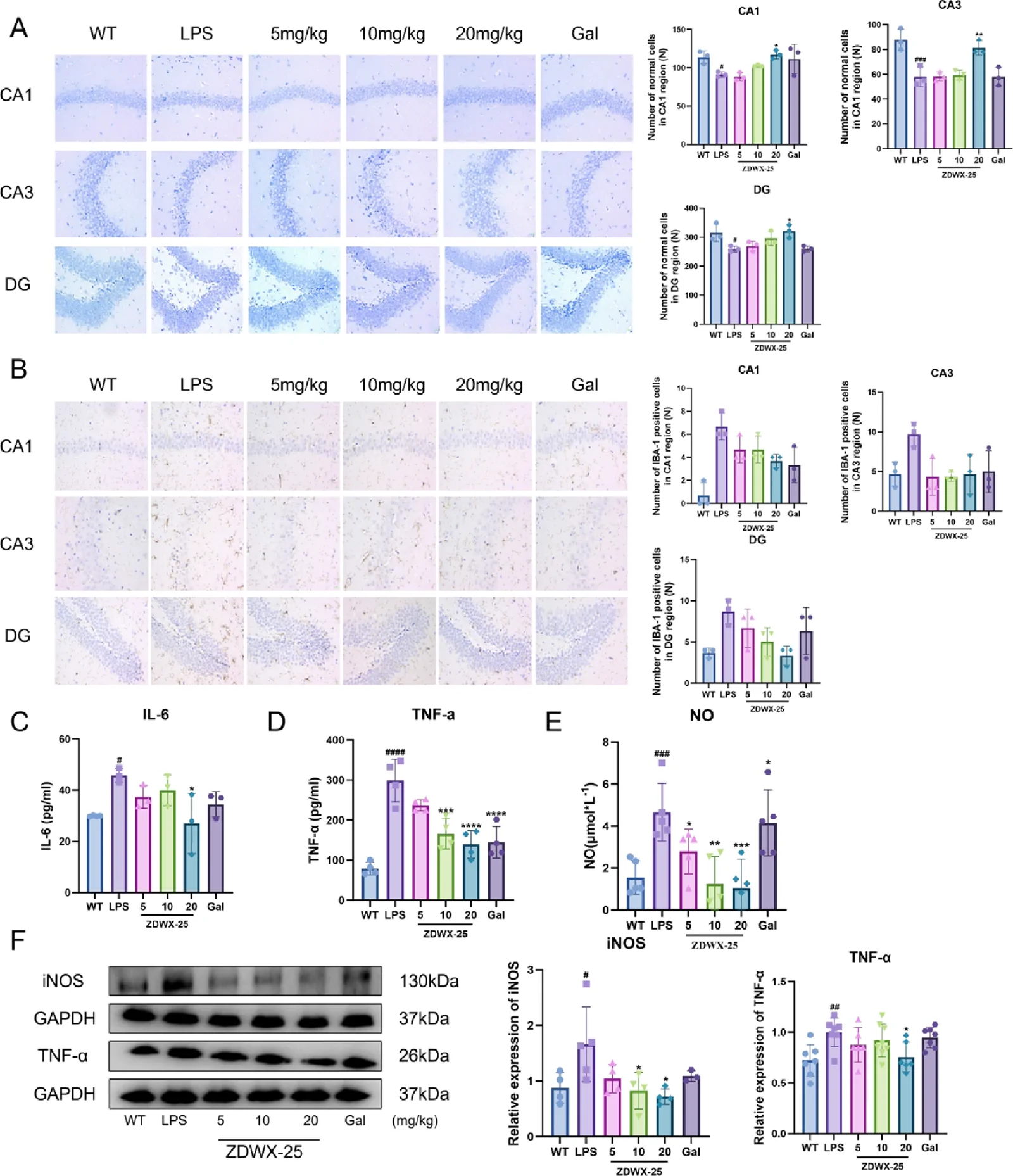
ZDWX-25 ameliorates LPS-induced neuronal injury and microglial activation in neuroinflammatory mice while reducing the expression of inflammatory factors in the brain. **(A)** Nissl staining of the mouse CA1, CA3, and DG regions. (Scale bar = 50 μm) **(B)** IBA-1 immunohistochemistry in the mouse CA1, CA3, and DG regions. (Scale bar = 50 μm). **(C,D)** Expression of the inflammatory cytokines IL-6 and TNF-α in the mouse brain. **(E)** NO levels in mouse plasma. **(F)** Protein expression of iNOS and TNF-α in mouse brain tissue. ^#^p < 0.05, ^##^p < 0.01, ^###^p < 0.001, ^####^p < 0.0001 vs. the control group; ^*^p < 0.05, ^**^p < 0.01, ^***^p < 0.001 vs. the model group. (n = 3–5 mean ± SEM). This figure presents a composite of Western blot bands from the same experiment and sample set that were run in parallel on separate gels and individually exposed. All bands represent parallel experimental results from the same batch of samples and have been uniformly processed and compared.

Hippocampal neuronal integrity was assessed via Nissl staining. LPS challenge induced severe neuronal loss and disordered architecture in the CA1, CA3, and DG regions. Treatment with ZDWX-25 (20 mg/kg) markedly increased the density of surviving neurons and ameliorated this histopathological damage (Figure 7A), demonstrating its neuroprotective effect against neuroinflammation.

Concurrently, microglial activation in the neuroinflammation model was assessed by IBA-1 immunohistochemistry to identify activated microglia. The hippocampus of the LPS-treated mice presented a significant increase in the number of Iba-1-positive cells, which typically displayed an amoeboid, activated morphology. This microglial activation was significantly suppressed by ZDWX-25 (20 mg/kg) treatment (Figure 7B).

To elucidate the molecular mechanisms underlying these protective effects, we measured the levels of key inflammatory mediators. ELISA and Western blot analysis of brain tissue revealed that LPS stimulation significantly increased the levels of IL-6, TNF-α, NO, and the protein iNOS. Treatment with ZDWX-25 (20 mg/kg) markedly suppressed the expression of these inflammatory factors (Figures 7C–F). These findings collectively demonstrate that ZDWX-25 exerts its potent anti-neuroinflammatory activity *in vivo* by concurrently mitigating neuronal damage, suppressing microglial activation, and dampening the production of proinflammatory cytokines.

### 3.7 ZDWX-25 attenuates neuroinflammation via dual inhibition of DYRK1A/GSK-3β and the downstream IκBα/NF-κB pathway

**FIGURE 8:**
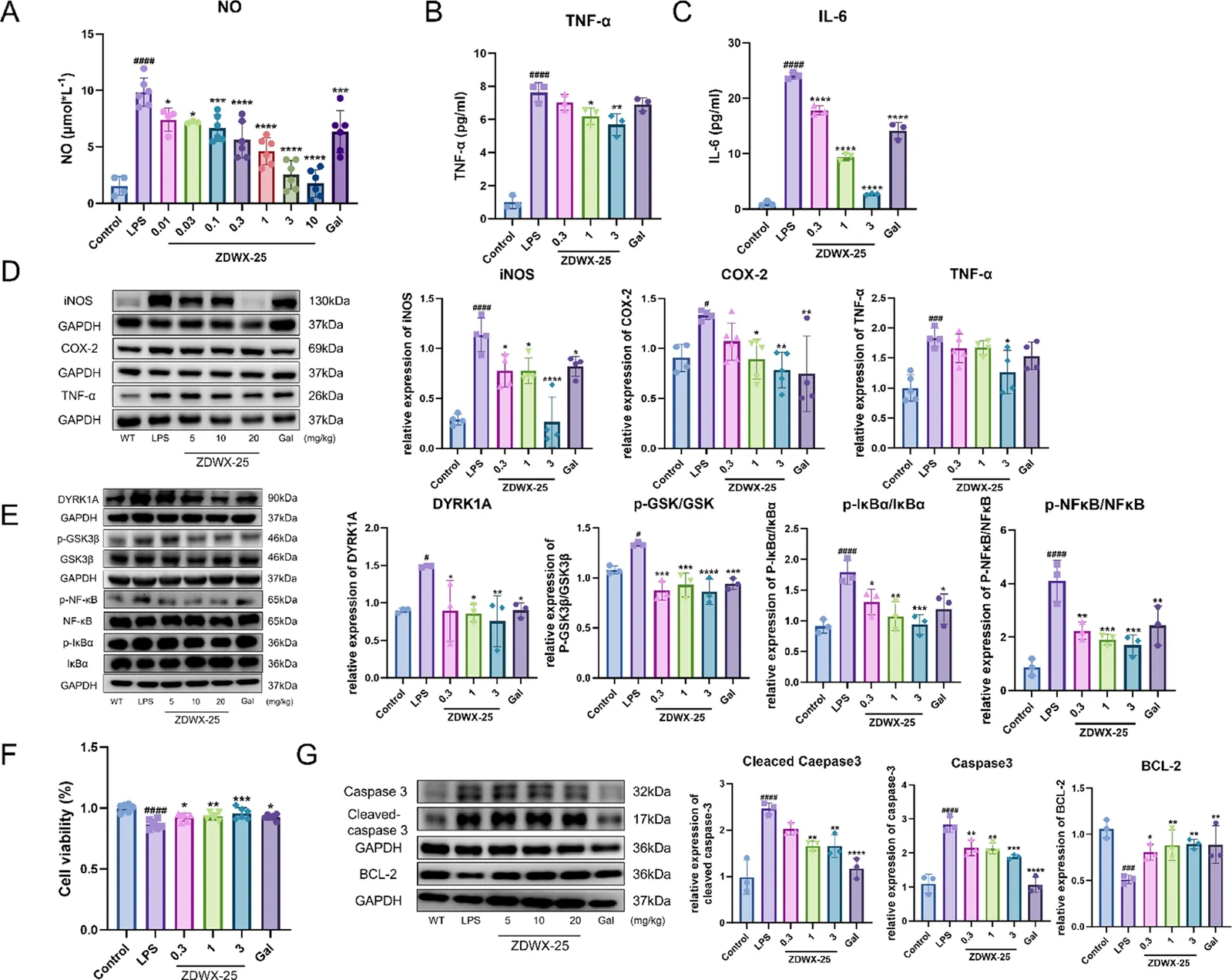
ZDWX-25 reduces LPS-induced inflammatory factor expression in BV2 cells and attenuates LPS-conditioned medium-induced apoptosis in HT22 cells. **(A)** ZDWX-25 dose-dependently reduces NO release. **(B)** ZDWX-25 reduces the LPS-induced increase in TNF-α levels in BV2 cells. **(C)** ZDWX-25 reduces the LPS-induced increase in IL-6 levels in BV2 cells. **(D)** ZDWX-25 reduces the expression of the inflammatory proteins iNOS, COX-2, and TNF-α in LPS-induced BV2 cells. **(E)** ZDWX-25 inhibits LPS-induced DYRK1A and GSK-3β target expression and inhibits IκBα/NFκB activation in LPS-treated BV2 cells. **(F)** ZDWX-25 reduced BV2 -conditioned medium-induced death in HT22 cells. **(G)** ZDWX-25 reduced the protein expression of cleaved caspase3, caspase3, and BCL2 in BV2 conditioned medium-treated HT22 cells. ^#^p < 0.05, ^##^p < 0.01, ^###^p < 0.001, ^####^p < 0.0001 vs. the control group; ^*^p < 0.05, ^**^p < 0.01, ^***^p < 0.001 vs. the model group. (n = 5 mean ± SEM). This figure presents a composite of Western blot bands from the same experiment and sample set that were run in parallel on separate gels and individually exposed. All bands represent parallel experimental results from the same batch of samples and have been uniformly processed and compared.

We next elucidated the mechanism *in vitro* via the use of LPS-stimulated BV2 microglia. The Griess assay confirmed that ZDWX-25 inhibited LPS-induced NO production in a dose-dependent manner, with significant effects observed even at 0.01 μM (Figure 8A). The ELISA results demonstrated that ZDWX-25 (3 μM) significantly suppressed the LPS-induced secretion of TNF-α and IL-6 (Figures 8B,C). Western blot analysis consistently revealed that ZDWX-25 downregulated the protein expression of iNOS, COX-2, and TNF-α (Figure 8D).

We next investigated whether the anti-inflammatory effects of ZDWX-25 were mediated through the downstream convergence point of DYRK1A and GSK-3β, the NF-κB pathway. As shown in Figure 8E, ZDWX-25 treatment potently inhibited the LPS-induced phosphorylation of IκBα and NF-κB p65. Given that ZDWX-25 concurrently reduced the activity of both DYRK1A and GSK-3β (Figure 8E), these results demonstrate that its dual kinase inhibition effectively disrupted the activation of the core proinflammatory IκBα/NF-κB signaling cascade.

### 3.8 ZDWX-25 reduces BV2-conditioned medium-induced apoptosis in HT22 cells

To assess the functional neuroprotective outcome of reduced neuroinflammation, HT22 cells were treated with conditioned medium (CM) from BV2 cells. CM from LPS-stimulated BV2 cells (Model CM) significantly reduced HT22 cell viability. In contrast, CM from BV2 cells pretreated with ZDWX-25 during LPS stimulation significantly increased neuronal survival (Figure 8F).

At the molecular level, Model CM upregulated the expression of the proapoptotic proteins caspase-3 and cleaved caspase-3 while downregulating the antiapoptotic protein Bcl-2 in HT22 cells. Treatment with ZDWX-25 (3 μM) reversed these changes in the expression of these apoptotic proteins (Figure 8G). These data demonstrate that ZDWX-25 mitigates microglial inflammatory responses, thereby reducing their neurotoxic effects and protecting neurons via an antiapoptotic mechanism.

## 4 Discussion

Current therapeutic strategies for Alzheimer’s disease (AD) are increasingly focused on tau pathology and neuroinflammation, reflecting the limited clinical efficacy of Aβ-targeting therapies (Long and Holtzman, 2019). In this study, we primarily used an OKA-induced acute tauopathy model to dissect the anti-tau mechanism of the dual DYRK1A/GSK3β inhibitor ZDWX-25, and secondarily explored its potential anti-inflammatory effects in an LPS model. A key rationale for selecting the OKA model is its direct relevance to our targets. OKA, a selective inhibitor of protein phosphatase PP2A, is known to simultaneously activate GSK3β and DYRK1A during tau hyperphosphorylation (Martin et al., 2011); GSK3β plays a critical role, while DYRK1A also contributes to phosphorylation at specific tau sites. Moreover, in OKA-treated neurons, GSK3β exhibits a paradoxical state of “maintained kinase activity despite increased inhibitory phosphorylation” (Lim et al., 2010; Chou and Yang, 2021), which limits the efficacy of single-target GSK3β inhibitors and favours a dual-target strategy. Consequently, the OKA model has been established as a valuable pharmacological tool for investigating AD-related tau pathology and neurotoxicity (Kamat et al., 2014). Its rapid induction enables efficient mechanistic dissection of downstream signalling pathways (e.g., PI3K/AKT/GSK-3β). Importantly, the core efficacy of ZDWX-25 has been independently validated in a chronic 3xTg AD mouse model (Liu et al., 2023); thus, the present study focuses on in-depth mechanistic exploration in an acute OKA tauopathy model. The key behavioural finding is that ZDWX-25 reversed spatial learning and working memory deficits in the OKA model, as assessed by both the Morris water maze and the Y-maze.

A key mechanistic insight from our study is that ZDWX-25 inhibits GSK3β through two convergent pathways: a direct, PI3K/AKT-independent inhibition, and an indirect, PI3K/AKT-dependent enhancement. Evidence for the direct pathway comes from HT22 cell experiments: the PI3K inhibitor LY294002 only partially reduced the effect of ZDWX-25, whereas the single-target GSK3β inhibitor AR-A014418 lost all efficacy (Figures 3E,F). This partial resistance indicates that ZDWX-25 possesses a direct GSK3β inhibitory activity that is absent in AR-A014418 under the same conditions. The indirect pathway is mediated by DYRK1A inhibition: harmine (a DYRK1A inhibitor) activated PI3K/AKT and increased inhibitory GSK3β Ser9 phosphorylation, an effect fully reversed by LY294002. Thus, by inhibiting DYRK1A, ZDWX-25 directly reduces tau phosphorylation (independently of PI3K/AKT) and simultaneously upregulates PI3K/AKT signalling to further suppress GSK3β. This dual mechanism allows ZDWX-25 to retain partial efficacy when the PI3K/AKT pathway is compromised, whereas single-target GSK3β inhibitors become completely ineffective–explaining the superiority of ZDWX-25 in reducing tau pathology, a finding we have previously validated in chronic 3xTg AD mice (Liu et al., 2023).

Another significant advancement is the elucidation of the capacity of ZDWX-25 to augment microglial phagocytic function and inhibit tau propagation, a mechanism distinct from that of most kinase inhibitors that act primarily within neurons. The propagation of pathological tau is a central mechanism in AD progression and is accelerated by impaired microglial phagocytic function (Bolós et al., 2016). Microglia can phagocytose extracellular phosphorylated tau (Scheiblich et al., 2024), but aberrant protein-mediated overactivation of microglia promotes tau pathology (Luo et al., 2015). In AD, this balance between phagocytosis and activation is disrupted (Sengupta and Kayed, 2022). Our findings indicate that ZDWX-25 not only enhances microglial phagocytosis of NFTs but also, crucially, significantly inhibits the subsequent propagation of pathological tau to neurons. This effect similarly relies on the PI3K/AKT signalling pathway. Notably, this dependence is absolute in microglia, as the PI3K inhibitor LY294002 completely blocked the pro-phagocytic effect of ZDWX-25 (Figures 3E,F, 5A–C). In contrast, in neurons, the anti-tau effect of ZDWX-25 was only partially dependent on PI3K/AKT (Figures 3E,F). This cell-type-specific requirement is consistent with the established critical role of PI3K/AKT signalling in microglial phagocytosis, for instance in TREM2-mediated clearance of pathological proteins (Ulland et al., 2017; Wang et al., 2020). Thus, ZDWX-25 employs a dual-cell mechanism in tau pathology: directly reducing p-tau within neurons while enhancing tau pathology suppression by amplifying microglial clearance functions. This approach overcomes the limitations of previous kinase inhibitors and aligns with the promising therapeutic strategy of modulating rather than broadly inhibiting microglial activity (Tang and Le, 2016).

In the LPS-induced neuroinflammatory model, intraperitoneal injection of lipopolysaccharide is a well-established paradigm to induce acute neuroinflammation and memory impairment, widely used for preclinical evaluation of candidate compounds against AD (Skrzypczak-Wiercioch and Salat, 2022). In our experiments, ZDWX-25 treatment improved MWM performance, and swimming speed analysis during the visible platform period showed no differences among groups, ruling out general motor dysfunction. However, we acknowledge several limitations in this part of the study: MWM performance may be influenced by sickness behaviour (e.g., lethargy, reduced motivation); non-stressful behavioural tests (e.g., Y-maze) were not performed; and body weight was not monitored. This study represents an initial exploration of the potential anti-neuroinflammatory effects of ZDWX-25. Our findings preliminarily suggest that ZDWX-25 possesses anti-neuroinflammatory activity that may contribute to cognitive improvement, an effect likely mediated by the dual inhibition of DYRK1A/GSK3β and subsequent modulation of the NF-κB/IκBα signalling pathway. Future studies should incorporate non-stressful behavioural paradigms (e.g., Y-maze or novel object recognition) to further validate these observations.

The dual-target strategy embodied by ZDWX-25 presents a compelling therapeutic advantage for complex diseases such as AD. By simultaneously engaging DYRK1A and GSK3β, ZDWX-25 orchestrates a coordinated attack on tau pathology and, based on our exploratory data, may also confer anti-neuroinflammatory benefits. Nevertheless, several limitations warrant consideration. First, the animal models used are acute and do not fully recapitulate the chronic, progressive neurodegeneration of human AD. Nonetheless, the mechanistic insights gained here directly explain the cognitive benefits previously observed by our group in chronic 3xTg mice (Liu et al., 2023). Second, only male mice were used in the present acute models; our prior work in female 3xTg mice confirmed efficacy in females, but future studies should include both sexes. Third, the conditioned medium experiments lacked a cell-free, drug-containing blank control to formally exclude direct drug carryover; however, the complete loss of protection by LY294002 in BV2 cells (Figure 5) strongly argues that the effect is microglia-mediated. Fourth, we did not use phosphorylation-site-specific antibodies to distinguish the contributions of DYRK1A versus GSK3β, a point to be addressed in future studies. Finally, while we have demonstrated the dual direct/indirect mechanism using pharmacological inhibitors, the precise molecular link by which DYRK1A inhibition leads to PI3K/AKT activation remains to be elucidated.

## 5 Conclusion

In conclusion, the cognitive-enhancing effects of ZDWX-25 are underpinned by a dual-cell, multipathway mechanism. In neurons, it restores cognitive function by attenuating tau hyperphosphorylation via DYRK1A-driven activation of the PI3K/AKT/GSK-3β axis. Concurrently, it promotes cognitive recovery by enhancing microglial phagocytic clearance of pathological tau and, in a neuroinflammatory setting, by potently suppressing the DYRK1A/GSK-3β/NF-κB pathway to prevent neuroinflammation. This multifaceted action successfully disrupts the vicious cycle between pathological tau propagation and neuroinflammation, representing a novel comprehensive therapeutic approach superior to single-target strategies.

## Data Availability

The original contributions presented in the study are included in the article/supplementary material, further inquiries can be directed to the corresponding authors.
